# Clinical symptoms related to anal sphincter defects and atrophy on external phased-array MR imaging

**DOI:** 10.1007/s00192-015-2743-4

**Published:** 2015-06-04

**Authors:** Imke Maria Henricus Kessels, Jurgen Jacobus Fütterer, Abdul Hameed Sultan, Kirsten Birgit Kluivers

**Affiliations:** 791 Department of Obstetrics and Gynaecology, Radboud University Medical Centre Nijmegen, P.O. 9101, 6500 HB Nijmegen, The Netherlands; Department of Radiology, Radboud University Medical Centre Nijmegen, P.O. 9101, 6500 HB Nijmegen, The Netherlands; Department of Obstetrics and Gynaecology, Croydon University Hospital, Surrey, UK

**Keywords:** Anal sphincter pathology, Clinical characteristics, Defecatory symptoms, Magnetic resonance imaging

## Abstract

**Introduction and hypothesis:**

Defecatory complaints have a severe impact on quality of life. The additional value of pelvic floor MRI in patients with defecatory complaints is unclear. Our aim was to correlate the presence of defects and atrophy of the anal sphincter complex using pelvic floor MRI in women with mixed pelvic floor symptoms and to establish patient characteristics and self reported complaints predictive of pathology.

**Methods:**

This is a retrospective study among women with mixed pelvic floor symptoms who underwent external phased-array MRI and completed a questionnaire on bothersome defecatory complaints. Data on patient characteristics, including obstetrical history and questionnaire scores were correlated with the assessment of anal sphincter defects and atrophy on pelvic floor MRI.

**Results:**

One hundred and fifty-eight women were included. A defect of the external anal sphincter (EAS) and internal anal sphincter (IAS) was found in 18 (11 %) and 5 (3 %) patients respectively. Atrophy of the EAS was present in 72 patients (46 %), with more cases of mild (*n* = 52, 33 %) than severe atrophy (*n* = 20, 13 %). The variable “previous third or fourth degree tear” had a significant positive association with an IAS defect on MRI, with an OR of 9.533 (1.425–63.776). Patients with EAS atrophy had higher scores for fecal incontinence (indicating more bother) than patients without EAS atrophy. Higher age and BMI were true predictors of the presence of more severe EAS atrophy.

**Conclusion:**

Atrophy of the EAS was highly prevalent in this population and was associated with bothersome symptoms of fecal incontinence.

## Introduction

A defect in the anal sphincter complex sustained during childbirth may cause fecal incontinence [1, 2]. Anal sphincter atrophy has more recently been identified as an explanation for late-onset fecal incontinence in the absence of an anal sphincter defect [3]. It is believed that anal sphincter atrophy may be related to pudendal neuropathy sustained because of stretching during childbirth [3–10].

Detecting anal sphincter atrophy in patients with fecal incontinence is clinically relevant because it is a negative predictive factor for the outcome of secondary anal sphincter repair [11]. In clinical practice, endoanal ultrasound and endoanal magnetic resonance imaging (MRI) are the main imaging modalities for the anatomical assessment of the anal sphincter complex [9].

External phased-array MRI of the anal sphincter complex is another imaging modality for delineating pathological conditions of the anal sphincter complex [5, 12–14]. This non-invasive imaging technique is more widely available than endoanal MRI and is less uncomfortable for the patient than any endoluminal imaging technique [13]. The limited data that exist on the comparison of external phased-array MRI with other imaging techniques, showed that external phased-array MRI was comparable with endoanal MRI in depicting clinically relevant anal sphincter defects and atrophy in fecally incontinent patients [5, 13, 14].

Available studies on the evaluation of the anal sphincter complex on external phased-array MRI were conducted in patients with complaints of fecal incontinence and were aimed at comparing mutual imaging techniques without comparison with functional assessment. Until now, the presence of anal sphincter defects and atrophy on external phased-array MRI has to our knowledge not been related to self-reported pelvic floor symptoms, e.g., defecatory complaints, in another population.

The aim of this study was to study whether any defecatory symptoms predict the presence and severity of defects and atrophy of the anal sphincter complex using external phased-array MRI in a general urogynecological population.

## Materials and methods

The data for this study were retrospectively collected as part of a large observational clinical cohort study on the pelvic floor. The study was carried out among women who visited the urogynecology outpatient clinics at the Department of Obstetrics and Gynecology of the Radboud University Medical Centre Nijmegen in the period 2005–2008. Patients who had completed the disease-specific quality-of-life questionnaire and had undergone external phased-array MRI were included. Patients were generally referred for external phased-array MR imaging of the pelvic floor as part of routine clinical practice in the case of recurrent POP, or in the case of a discrepancy between clinical signs and symptoms of pelvic floor dysfunction.

Exclusion criteria were: vaginal nulliparity, any kind of pelvic floor surgery between MRI and completion of the questionnaire, and/or more than 1 year between the completion of the questionnaire and the MRI. This cohort study was submitted and officially deemed exempt from local Institutional Review Board approval since all data were acquired as part of routine clinical practice and this study was regarded as a component of quality control.

Patients completed a questionnaire on their general health and obstetrical history, designed by the working party on pelvic floor and urogynecology of the Dutch Society of Obstetrics and Gynecology. This questionnaire included mode(s) of delivery, including the use of vacuum extraction and forceps, and the presence of an episiotomy and/or an obstetrical tear. Furthermore, patients were asked to enter their height, weight and any co-morbidities. The presence of a third or fourth degree tear, mode of repair, and data on general health (including postmenopausal status and diabetes) were additionally collected from the medical files.

In the case of missing data in patients older than 57 years, the assumption was made that they were of postmenopausal status [15].

The Dutch version of the Defecatory Distress Inventory (DDI) [16] was used to measure defecatory symptoms as well as the degree of bother. This questionnaire consists of ten items, grouped over five subscales. Each subscale score ranges from 0 to 100, where 0 indicates the best quality of life (no symptoms present or symptoms present but causing no bother) and 100 indicates that all symptoms are present and causing maximal bother. The subscale score is calculated as the mean of the corresponding item scores.

### MR imaging

Magnetic resonance imaging was performed with the patient in the supine position. Patient preparation consisted of 10 mg of bisacodyl orally administered the day before the examination, 1 mL of butylscopolamine was injected intramuscularly before imaging. Patients were asked to empty their bladder before imaging. A three-dimensional (3D) T2-weighted (1 × 1 × 1 mm^3^ resolution) MR sequence and a high in-plane resolution axial T2-weighted turbo spin echo (0.5 × 0.5 × 3 mm^3^, no slice gap) MR sequence were applied.

### Image analysis

All images were analyzed for the presence and severity of defects (including scars) and atrophy of the internal anal sphincter (IAS) and external anal sphincter (EAS). Measurements of the anal sphincter complex were also taken. The MRI datasets were reviewed by an experienced pelvic floor radiologist (JF, 5 years’ experience). The observer was blinded to all clinical findings, except for age, since the birth dates were visible on the scans. Images were imported and analyzed in a 3D imaging post-processing program (TeraRecon, San Mateo, CA, USA), which enabled the observer to reconstruct the 3D data sets to obtain images aligned with the puborectal sling.

**Fig. 1:**
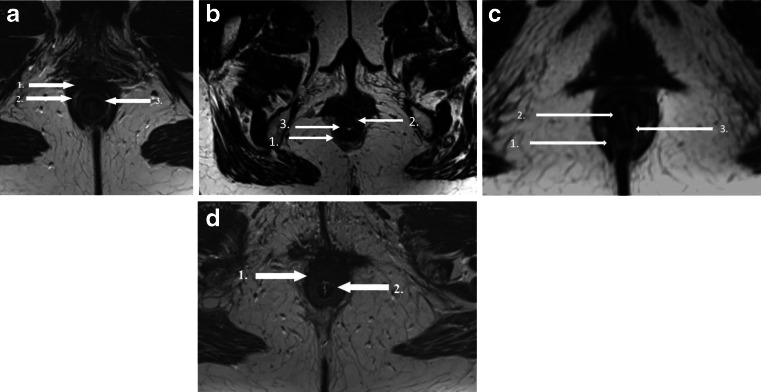
**a** Axial T2-weighted turbo spin-echo MRI showing a normal external and internal sphincter complex in a 42-year-old woman.* 1* external anal sphincter (EAS);* 2*  conjoint longitudinal coat,* 3* internal anal sphincter. (IAS) **b** Axial T2-weighted turbo spin-echo MRI showing a defect (defined as a discontinuity of the muscle ring [either isolated or combined, anatomical defect] and/or a hypointense deformation of the muscle ring owing to the replacement of muscle cells by fibrous tissue [functional defect, scar tissue]) of the EAS in a 67-year-old woman. There is also some EAS atrophy.* 1* EAS;* 2*  EAS defect;* 3*  IAS. **c** Axial T2-weighted turbo spin-echo MRI showing a combined IAS and EAS defect defined as a discontinuity of the muscle ring (either isolated or combined, anatomical defect) and/or a hypointense deformation of the muscle ring owing to the replacement of muscle cells by fibrous tissue (functional defect, scar tissue) in a 31-year-old woman.* 1* EAS;* 2* combined IAS and EAS defect;* 3* internal anal sphincter. **d** Axial T2-weighted turbo spin-echo MRI showing severe atrophy of the external anal sphincter muscle (≥ 50 % thinning or replacement of the sphincter muscle by fat) in a 67-year-old woman. There is some IAS hypertrophy.* 1*  EAS;* 2*  IAS

In the axial plane on MRI, both the IAS and EAS can be seen as a clearly defined ring with relatively hyperintense signal intensity for the innermost IAS and relatively hypointense signal intensity for the outermost EAS. [17]. At the midanal level the EAS appears as a typical teardrop, with the tip of the drop pointing to the coccyx (Fig. 1a).

An IAS or EAS defect was defined as a minimum of 30° discontinuity of the muscle ring (either isolated or combined; anatomical defect) and/or was recognized by a hypointense deformation of the muscle ring owing to the replacement of muscle cells by fibrous tissue (functional defect, scar tissue; Fig. 1b, c) [4].

The following grading system proposed by Terra et al. [5], was used to stratify EAS atrophy by means of the percentage of fat content of the EAS and measurements of the area of the remaining EAS:

1. No atrophy = no thinning of replacement of the sphincter muscle by fat
2. Mild atrophy = < 50 % thinning of replacement of sphincter muscle by fat
3. Severe atrophy = > 50 % thinning of replacement of sphincter muscle by fat

Figure 1d shows an EAS with severe atrophy.

The EAS was also measured at two predetermined points on the axial image: at the point where it had the thickest volume and at the 9 o’clock position (right lateral side). When fatty replacement had occurred within the EAS, the thickness at the 9 o’clock position and the thickest point included not only muscle cells but also fat mixed in the EAS. Measurements were not taken of the anterior aspect of the sphincter complex, because of the possible distortion from obstetric anal sphincter injury [1, 2, 18].

**Fig. 2:**
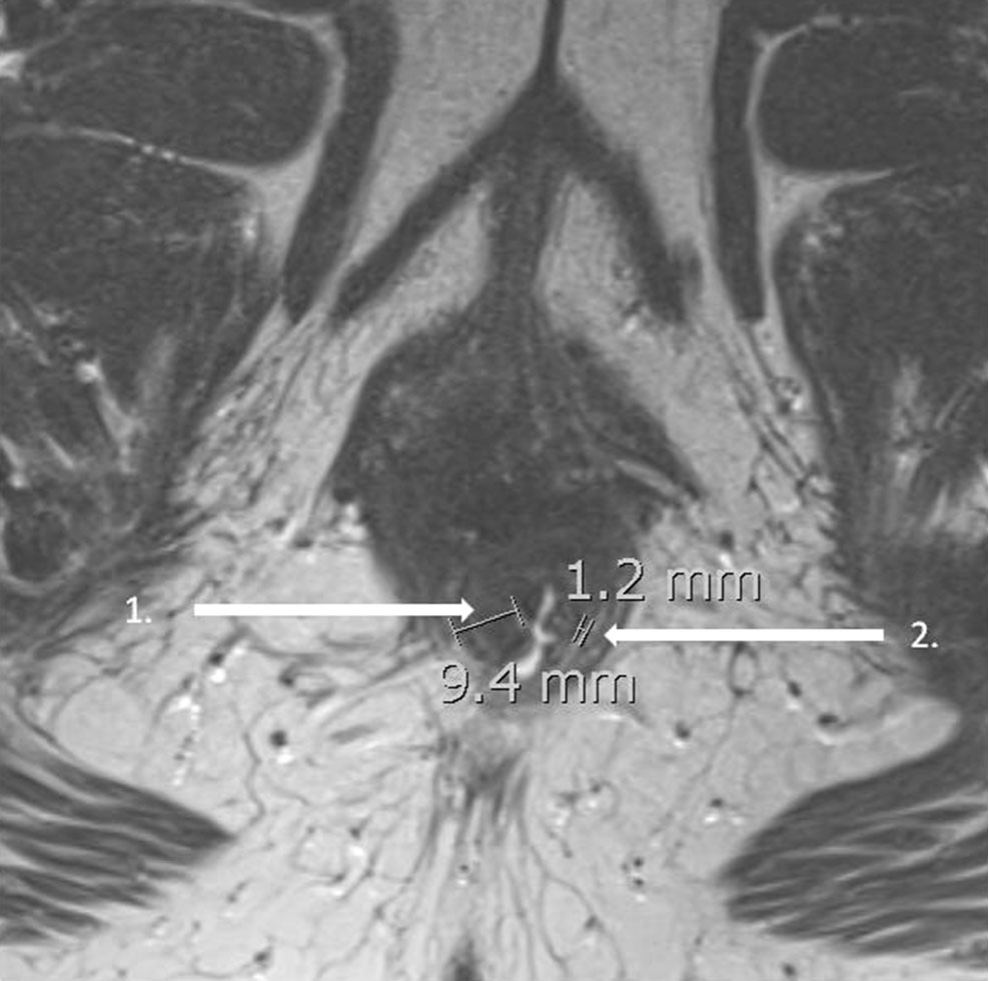
Axial T2-weighted turbo spin-echo MRI showing anal sphincter measurements in a 49-year-old woman.* 1* total sphincter thickness (9.4 mm);* 2* hyperintense intersphincteric fat (1.2 mm)

Atrophy of the IAS was defined as a severe thinning of the internal anal muscle (<2 mm) [19]. The total sphincter thickness (in millimeters) was measured from the inner border of the IAS to the outer border of the EAS. In addition, the number of millimeters of hyperintense intersphincteric fat was determined (Fig. 2).

The observers evaluated the identification of the IAS and EAS as being adequate (moderate-to-good) or inadequate (poor) on the basis of the possibility of evaluating the separate structures of the IAS and EAS. The presence of artifacts was scored and the overall quality of the MR image was recorded as adequate (moderate-to-good) or inadequate (poor). Only the images with adequate identification of both IAS and EAS and adequate overall quality were included in this study.

### Statistics

To compare continuous variables in the EAS and IAS defect groups, unpaired* t* tests (age and BMI) and Mann–Whitney* U* tests (questionnaire subscale scores) were used. One-way analysis of variance (ANOVA) was used to compare continuous variables among the three groups of EAS atrophy. In the case of a statistically significant difference, the post hoc least significant difference procedure was applied for intergroup comparison. To identify differences between groups in the case of binary variables, Fisher’s exact test was used.

The following variables were used as independent variables in the univariate logistic regression analyses: age, body mass index (BMI), episiotomy, third or fourth degree tear, instrumental delivery, presence of diabetes, postmenopausal status, and questionnaire subscale scores. Defects of the EAS and IAS, and atrophy of the EAS based on MRI were used as separate dependent variables.

To identify potential risk factors for pathological conditions of the anal sphincter and to calculate odds ratios (OR), multivariate logistic regression analysis with manual backward elimination was applied. On the dependent variable “EAS atrophy,” multivariate ordinal logistic regression was applied.

The choice of multivariate ordinal logistic regression instead of multinomial logistic regression was made based upon the fact that in multivariate ordinal logistic regression analysis, valuable information on the severity of EAS atrophy was retained. All variables with a* p* value < 0.20 were entered into the multivariate ordinal logistic regression analysis. Analyses were performed using statistical software (SPSS 20.0 for Windows; SPSS, Chicago, IL, USA). *p* values < 0.05 were considered to indicate statistical significance.

## Results

A total of 190 patients underwent MRI and completed the questionnaire between 2005 and 2008. Five datasets were excluded because the MR images were of poor quality; 7 women were vaginally nulliparous. In 11 cases, there were more than 365 days or a pelvic surgical procedure was performed between imaging and completion of the questionnaire. Date of completion of the questionnaire was unclear in 9 cases, which also led to exclusion. Four MR datasets had severe motion artifacts, but the overall quality of the images was sufficient to be included. Therefore, the data of 158 patients were available for statistical analysis.

The prevalence of pathological conditions of the IAS and EAS on external phased-array MRI is shown in Table 1.

**Table 1:** Prevalence of defects and atrophy of the external anal sphincter (EAS) and internal anal sphincter (IAS) on external phased-array MRI

	Number (% of patients), total *N* = 158
Completely normal sphincter complex	76 (48)
Defect EAS	18 (11)
Solitary defect EAS	14 (9)
Defect IAS	5 (3)
Solitary defect IAS	1 (1)
Combined defect EAS and IAS	4 (3)
EAS atrophy	72 (46)
Mild	52 (33)
Severe	20 (13)
IAS atrophy	0 (0)

No cases of IAS atrophy were identified.

### EAS and IAS defects

There were no differences in baseline characteristics observed between patients with and without an EAS defect (Table 2). Compared with patients without an IAS defect, those with an IAS defect sustained significantly more third or fourth degree obstetric tears (*p* = 0.047; 6.5 vs 40 %; Table 3). Furthermore, compared with patients with an IAS defect, significantly more patients without an IAS defect were postmenopausal (*p* = 0.019; 0 vs 87.5 %). However, these groups were very small. One patient underwent a lateral internal anal sphincterotomy: MRI showed no defect of the IAS; however, mild atrophy of the EAS was seen. Two patients underwent sphincter repair later in life (i.e., not immediately after childbirth): both patients showed no defect or scarring of the EAS; mild atrophy was, however, present in both patients.

**Table 2:** Patient characteristics of women with and without EAS defects on external phased-array MRI

	No EAS defect (*n* = 140)		EAS defect (*n* = 18)		*p* value^a^	*p* value^b^
	*n*	Mean or* n* (%)	*n*	Mean or* n* (%)		
Age (years)	140	55.3	18	57.4	0.43	0.43
BMI (kg/m^2^)	129	25.9	17	25.5	0.70	0.7
Obstetric history						
Episiotomy	130	9 (6.4) 91 (70.0)	18	12 (66.7)	0.79	0.77
3rd/4th degree tear	140		18	3 (16.7)	0.14	0.14
Instrumental delivery	133	15 (11.3)	18	1 (5.6)	0.69	0.47
Diabetes	140	5 (3.6)	18	0 (0.0)	1.00	1.00
Postmenopausal status	100	87 (87.0)	14	11 (78.6)	0.41	0.40

*BMI* body mass index, *Instrumental delivery* vacuum-assisted delivery or forceps delivery

^a^ *p* value for difference between groups using unpaired* t* tests and Fisher’s exact test, as appropriate

^b^ *p* value on univariate logistic regression analysis

**Table 3:** Patient characteristics and Defecatory Distress Inventory (DDI) subscale scores of women with and without IAS defects on external phased-array MRI

	No IAS defect (*n* = 153)		IAS defect (*n* = 5)		*p* value^b^	*p* value^c^
	*n*	Mean or* n* (%)	n	Mean or* n* (%)		
Age (years) ^a^	153	55.8	5	47.6	0.08	0.09
BMI (kg/m^2)^	141	25.9	5	23.9	0.25	0.25
Obstetric history						
Episiotomy	143	99 (69.2)	5	4 (80.0)	1.00	0.61
3rd/4th degree tear^a^	153	10 (6.5)	5	2 (40.0)	0.05	0.02
Instrumental delivery	146	16 (11.0)	5	0 (0.0)	1.00	1.00
Diabetes	153	5 (3.3)	5	0 (0.0)	1.00	1.00
Postmenopausal status	112	98 (87.5)	2	0 (0.0)	0.02	1.00

^a^Variable entered in multivariate analysis

^b^ *p* value for difference between groups using unpaired* t* tests and Fisher’s exact test, as appropriate

^c^ *p* value on univariate logistic regression analysis

In Tables 4 and 5, the subscale scores of the DDI for the EAS and IAS defect groups are depicted. On all subscale scores, a wide range was observed. The scores in the groups with and without an EAS defect were similar. Compared with patients without an IAS defect, higher scores on the subscales for obstructive defecation, fecal incontinence, and flatus incontinence were observed in patients with an IAS defect, but the differences were not statistically significant.

**Table 4:** Defecatory Distress Inventory (*DDI*) subscale scores of women with and without EAS defects on external phased-array MRI

DDI subscale scores	*n*	Median (30)	*n*	Median (30)	*p* value^a^	*p* value^b^
Constipation	139	16.7 (0–100)	18	16.7 (0–83.3)	0.91	0.98
Obstructive defecation	140	16.7 (0–100)	18	16.7 (0–83.3)	0.88	0.66
Pain	139	0 (0–100)	18	0 (0–66.7)	0.25	0.28
Fecal incontinence	139	0 (0–100)	17	0 (0–83.3)	0.93	0.89
Flatus incontinence	140	33.3 (0–100)	17	33.3 (0–100)	0.78	0.78

^a^ *p* value for difference between groups using Mann–Whitney* U* tests (asymptotic)

^b^ *p* value on univariate logistic regression analysis

**Table 5:** Defecatory Distress Inventory (*DDI*) subscale scores of women with and without IAS defects on external phased-array MRI

DDI subscale scores	*n*	Median (30)	*n*	Median (30)	*p* value^b^	*p* value^c^
Constipation	152	16.7 (0–100)	5	(0–33.3)	0.26	0.27
Obstructive defecation	153	16.7 (0–100)	5	25 (0–50)	0.88	0.62
Pain	152	0 (0–100)	5	0 (0–66.7)	0.36	0.51
Fecal incontinence	151	0 (0–100)	5	16.7 (0–50)	0.97	0.71
Flatus incontinence^a^	152	33.3 (0–100)	5	66.7 (0–100)	0.17	0.12

^a^Variable entered in multivariate analysis

^b^ *p* value for difference between groups using Mann–Whitney* U* tests (asymptotic)

^c^ *p* value on univariate logistic regression analysis

Univariate logistic regression analysis was performed for the EAS defect and IAS defect groups. Only the variable “third- or fourth-degree tear” had a significant positive association with an IAS defect on MRI, with an OR of 9.533 (1.425–63.776).

There was no multivariate logistic regression performed on the EAS defect group, because in the univariate analysis there were no factors identified with potential statistical significance. Multivariate logistic regression was performed on the IAS defect group on the variables with a *p* value < 0.20 (age, third- or fourth-degree tear, flatus incontinence). None of these variables was statistically significant (*p* values of 0.27, 0.12 and 0.25 respectively) in the multivariate analysis.

### EAS atrophy

Table 6 shows baseline characteristics in patients with no, mild, and severe EAS atrophy. Patients with EAS atrophy (mild or severe) were significantly older and had a higher BMI than patients without EAS atrophy. There were 5 patients with diabetes: all of them showed mild EAS atrophy.

**Table 6:** Patient characteristics of women with no, mild, and severe EAS atrophy on external phased-array MRI

	No EAS atrophy (*n* = 86)		Mild EAS atrophy (*n* = 52)		Severe EAS atrophy (*n* = 20)		*p* value ^b^	*p* value ^c^
	*n*	Mean or* n* (%)	*n*	Mean or* n* (%)	*n*	Mean or* n* (%)		
Age (years)^a^	86	52.8	52	57.5	20	62.5	Overall <0.01	< 0.01
							<0.01*	
							0.06**	
							<0.01***	
BMI (kg/m^2^)^a^	82	24.8	46	26.9	18	27.7	Overall <0.01	<0.01
							<0.01*	
							0.38**	
							0.01***	
Obstetrical history								
Episiotomy	79	54 (68.4)	50	38 (76.0)	19	11 (57.9)	0.32	0.95
3rd/4th degree tear	86	5 (5.8)	52	5 (9.6)	20	2 (10.0)	0.62	0.38
Instrumental delivery	84	6 (7.1)	48	8 (16.7)	19	2 (10.5)	0.21	0.23
Diabetes^a^	86	0 (0.0)	52	5 (9.6)	20	0 (0.0)	<0.01	0.19
Postmenopausal status	48	39 (81.3)	49	42 (85.7)	17	17 (100)	0.14	0.12

*Statistically significant difference between no atrophy and mild atrophy; **statistically significant difference between mild atrophy and severe atrophy; ***statistically significant difference between no atrophy and severe atrophy

^a^Variable entered into multivariate analysis

^b^ *p* value for difference between groups using analysis of variance with least significant difference post hoc adjustment for intergroup comparison and Fisher’s exact test, as appropriate

^c^ *p* value on univariate logistic regression analysis

The mean thickness of the EAS at the 9 o’clock position was 3.5 mm (range 1.0–8.9 mm, SD 1.4 mm), and at the thickest point it was 4.2 mm (range 1.5–8.9, SD 1.6 mm). The mean total thickness of the sphincter complex was 9.7 mm (range 5.0–18.7, SD 2.4) and the mean in intersphincteric fat was 2.7 mm (range 0–7.7 mm, SD 1.6 mm). There were no statistical differences present with regard to these four variables among patients with no, mild, and severe atrophy.

The subscale scores of the DDI for the EAS atrophy groups are shown in Table 7. Women with severe EAS atrophy scored significantly lower on the subscale “constipation” than patients with no atrophy. On the subscale “obstructive defecation,” women with mild atrophy scored significantly lower than patients with no atrophy. Finally, women with severe EAS atrophy scored significantly higher on the subscale “fecal incontinence” than patients with no or mild EAS atrophy.

**Table 7:** Defecatory Distress Inventory (DDI) subscale scores of women with no, mild, and severe external anal sphincter (EAS) atrophy on external phased-array MRI

DDI subscale scores	*n*	Median (30)	*n*	Median (30)	*n*	Median (30)	*p*-value^b^	*p* value ^c^
Constipation^a^	86	25 (0–100)	51	16.7 (0–100)	20	0 (0–83.3)	Overall 0.03	0.02
							0.15*	
							0.15**	
							0.02***	
Obstructive defecation^a^	86	25 (0–100)	52	16.7 (0–91.7)	20	16.7 (0–100)	Overall 0.07	<0.01
							0.02*	
							0.77**	
							0.29***	
Pain	86	0 (0–100)	51	16.7 (0–100)	20	0 (0–100)	Overall 0.62	0.57
							0.99*	
							0.31**	
							0.38***	
Fecal incontinence^a^	85	0 (0–83.3)	51	0 (0–100)	20	25 (0–100)	Overall 0.03	0.02
							0.82*	
							0.03**	
							<0.01***	
Flatus incontinence	85	33.3 (0–100)	52	33.3 (0–100)	20	33.3 (0–100)	Overall 0.21	0.54
							0.61*	
							0.07**	
							0.16***	

*Statistically significant difference between no atrophy and mild atrophy; **statistically significant difference between mild atrophy and severe atrophy; ***statistically significant difference between no atrophy and severe atrophy

^a^Variable entered into multivariate analysis

^b^ *p* value for differences between groups using the Kruskal–Wallis test, and comparing separate groups using separate Mann–Whitney* U* tests

^c^ *p* value on univariate logistic regression analysis

The variable “postmenopausal status” had a *p* value of <0.2 in the univariate logistic regression, but was removed from the multivariate (ordinal) logistic regression analysis because of too many missing variables. In the manual backward elimination, only age and BMI were significant predictors in the multivariate ordinal logistic regression model (*p* = 0.000 and *p* = 0.001). For age, the OR was 1.088 (95 % CI 1.047–1.130). This indicates that in a woman who was 10 years older, the OR for having severe EAS atrophy (versus no or mild atrophy) was 2.32 (1.088^10^). A similar effect was identified for BMI, with an OR 1.181 (95 % CI 1.074–1.301). For a woman with a 10 m^2^/kg higher BMI the OR of having severe EAS atrophy (versus no or mild atrophy) was 5.31.

## Discussion

External anal sphincter atrophy is the most common finding in this patient group, with age and BMI being true predictors of this condition. Patients with EAS atrophy scored higher on the questionnaire subscale “fecal incontinence.” Age and BMI were true predictors for the presence of EAS atrophy.

A limited number of studies have assessed the pathology conditions of the anal sphincter complex on external phased-array MRI [5, 13, 14, 20]. Overall, the number of defects of the EAS and IAS detected was lower in our study compared with other studies. In a study among 30 patients by Terra et al. [14], 33 % had an EAS defect and 6.7 % had an IAS defect compared with 11 and 3 % in the present study. In another study by Terra et al. [5] mild EAS atrophy was found in 17 %, severe EAS atrophy in 27 versus 33 and 13 % in the present study. Previous studies were performed in older subjects, with fecal incontinence and also included men. This female study population consisted of patients with a wide spectrum of complaints of pelvic floor dysfunction. This may also partly explain the difference found in the presence of defects and atrophy found [5, 14].

There are no comparable studies available that have correlated the image of the anal sphincter complex on external phased-array MRI with patient characteristics and questionnaire results. With regard to endoanal MR imaging, Terra et al. [21] have studied the relation between pelvic floor muscle lesions and the severity of fecal incontinence (assessed according to the Vaizey scale) in 105 women. No relationship was found between the mean Vaizey score and the presence of lesions in any part of the pelvic floor or anal sphincter. Studies on the relationship between (the size of) sphincter defects on ultrasound and the presence and severity of fecal incontinence are contradictory [22–24].

Since mechanical injury during vaginal delivery is the most common cause of an anal sphincter defect [25], patients with an IAS defect were significantly more likely to have sustained a third- or fourth-degree tear during childbirth. Moreover, the variable third- or fourth-degree tear had a significant positive association (only in the univariate analysis) with an IAS defect on MRI, with an OR of 9.533, but there were only few IAS defects (*n* = 5). Patients without an IAS defect were more likely to be postmenopausal than patients with an IAS defect. A possible explanation is that separate primary repair of the IAS was only described by Sultan in 1999 and therefore older women were less likely to have had the internal sphincter repaired at the time of childbirth [26].

We expected to find higher scores on the DDI subscale “fecal incontinence” in patients with an anal sphincter defect (EAS or IAS), compared with patients without defects. However, the differences were not statistically significant. Although sphincter defects are known to be related to fecal incontinence, we were not able to demonstrate this in the current patient group. This therefore suggests that other factors might play a role in the development of fecal incontinence.

Mild EAS atrophy may be more common than previously thought and can be found with external phased-array MR imaging. Women with severe EAS atrophy scored higher on the subscale ‘fecal incontinence’ than patients with no or mild EAS atrophy.

Patients with EAS atrophy (mild or severe) were significantly older and had a higher BMI than patients without EAS atrophy. When correcting for other variables, age and BMI appeared to be genuine predictors for (more severe) EAS atrophy. As far as aging is concerned, our findings are in accordance with those of previous literature, where an increase in the proportion of connective tissue in the EAS and a progressive reduction in EAS thickness caused by aging have been described [27, 28]. For BMI, this association has not been described previously. A higher fat percentage in the body may explain the presence of fat in the anal sphincter complex, leading to a potential overdiagnosis of EAS atrophy.

In all patients with diabetes mellitus mild EAS atrophy was seen. It has been described that diabetes can lead to fecal incontinence owing to autonomic neuropathy and an impaired anorectal sensation in the anal sphincter complex [25].

Terra et al. [5] have found a significant difference in EAS thickness at the right lateral side between patients with and without EAS atrophy. In our study, we did not find this difference, probably because the sphincter measurements included the intersphincteric fat. When fatty replacement has occurred within the EAS, the thickness at the 9 o’clock position and the thickest point includes muscle cells as well as interposed fat within the EAS.

The strength of this study was the large number of participants compared with other MRI studies on the anal sphincter complex. The clinical relevance of diagnosing EAS atrophy has been highlighted by Deutekom et al. [29], demonstrating that these women are more likely to have complaints of (urge) fecal incontinence. Furthermore, EAS atrophy is associated with a poorer outcome following secondary anal sphincter repair [11].

This study has the following potential limitations for consideration. Some data on possible risk factors for EAS atrophy, i.e., obstetrical details, were collected from patients’ memory rather than from patients’ records. The information on patients’ pre- or postmenopausal status was not always available. In the absence of data concerning pre- or postmenopausal status, the choice was made to score the patients older than 57 years as postmenopausal, since the chance that a woman is not postmenopausal at the age of 58 is only 5 % [15]. We used a self-reported questionnaire on defecatory complaints that has not been validated in detail. There were no strict criteria in the protocol for MRI requests and accurate subgroup analysis was not possible. Concerning the evaluation of the MR images, there was a visual subjective interpretation of thinning of the EAS and/or replacement of the EAS muscle by fat as there is no agreed classification for the visual diagnosis of EAS atrophy on MRI.

It is debatable whether an anatomical defect and functional defect (scar tissue) can be identified as two different entities. In this study defects and scars were analyzed as one group, because the clinical implications for these conditions are expected to be similar and it is very difficult to distinguish between these anatomical and functional defects on external phased-array MRI. Furthermore, there was no predetermined level in the longitudinal plane to measure and evaluate the sphincter complex. Terra et al. [14] have the level of 1 cm cephalad to the lower border of the EAS (also at the 9 o’clock right lateral position). In this study group, this level could often not be properly identified, because of varying anatomy due to pelvic organ prolapse. Therefore, the longitudinal level was used in which the characteristics of the sphincter complex could be seen satisfactorily.

In conclusion, atrophy of the EAS appears to be an underdiagnosed pathological condition that can be identified with external phased-array MRI. EAS atrophy is associated with unexplained defecatory complaints (especially in older patients with a higher BMI) and a suboptimal outcome of secondary anal sphincter repairs. It is controversial whether EAS atrophy can be reliably and consistently diagnosed on endoanal ultrasound. External phased-array MRI appears to be a good alternative to endoanal MRI, as it is less intrusive and uncomfortable for the patient and is more widely available.
